# Study of genetic polymorphisms and steady-state trough concentrations of imatinib and its active metabolite in predicting efficacy in gastrointestinal stromal tumors

**DOI:** 10.3389/fphar.2025.1604619

**Published:** 2025-05-19

**Authors:** Menghua Zhang, Zhiyao Chen, Xiaoxue Liu, Xiaojun Zhou, Liyan Miao

**Affiliations:** ^1^ Department of Pharmacy, The First Affiliated Hospital of Soochow University, Suzhou, Jiangsu, China; ^2^ Department of General Surgery, The First Affiliated Hospital of Soochow University, Suzhou, Jiangsu, China; ^3^ Institute for Interdisciplinary Drug Research and Translational Sciences, Soochow University, Suzhou, Jiangsu, China; ^4^ College of Pharmaceutical Sciences, Soochow University, Suzhou, Jiangsu, China; ^5^ National Clinical Research Center for Hematologic Diseases, The First Affiliated Hospital of Soochow University, Suzhou, Jiangsu, China

**Keywords:** gastrointestinal stromal tumors, imatinib, N-desmethyl imatinib, efficacy, steady-state trough concentration, genetic polymorphisms

## Abstract

The imatinib (IMA) steady-state trough concentration (C_min_) plays a critical role in the treatment outcomes of patients with gastrointestinal stromal tumors (GISTs), yet the effective concentration range in the Chinese population remains unclear. Additionally, few studies have investigated the effects of N-desmethyl imatinib (NDI) and genetic polymorphisms in metabolic enzymes and transporters on GIST treatment efficacy. Therefore, the aim of this study was to determine the value of the IMA and total (IMA + NDI) C_min_ for the prediction of treatment outcomes in advanced GIST patients and to assess the influence of genetic polymorphisms on the IMA and total (IMA + NDI) C_min_ and treatment efficacy. Twenty-one IMA-treated patients with advanced GIST were enrolled. An IMA C_min_ ≥950 ng/mL and an IMA + NDI C_min_ ≥956 ng/mL were associated with a reduced PD risk, with area under the receiver operating characteristic curve (AUC) values of 0.944 and 0.967, respectively. Higher IMA and IMA + NDI C_min_ and higher risks of PD were observed in C allele carriers of rs2231137 and A allele carriers of rs2725252 in *ABCG2* and in G allele carriers of rs2631372 in *SLC22A5.* In conclusion, the IMA and IMA + NDI C_min_ can serve as effective indicators of advanced GIST treatment outcomes. Drug efficacy should be monitored in patients with an IMA C_min_ <950 ng/mL or a total (IMA + NDI) C_min_ <956 ng/mL. Furthermore, genetic polymorphism testing is recommended before dosing to appropriately adjust the IMA dose for carriers of the C allele in rs2231137, the A allele in rs2725252 in *ABCG*2, and the G allele in rs2631372 in *SLC22A5*.

## 1 Introduction

Imatinib (IMA) is the first-line treatment for unresectable and metastatic gastrointestinal stromal tumors (GISTs) (Poveda et al., 2017) and significantly improves patient outcomes and prolongs survival (Cavnar et al., 2021; Heinrich et al., 2017; Raut et al., 2018; Reichardt, 2018). The steady-state trough concentration (C_min_) of the IMA considerably impacts clinical outcomes in patients with advanced GIST (Bouchet et al., 2016). Demetri et al. (2009) suggested that patients with advanced GIST experience a significantly shorter time to disease progression when the Cmin of the IMA falls below 1,110 ng/mL. N-desmethyl imatinib (NDI), the active metabolite of IMA, exhibits biological activity similar to that of IMA, with plasma concentrations reaching 20%–25% of that of the parent drug at steady state (Al-Hadiya et al., 2014; Delbaldo et al., 2006; Peng et al., 2005). It is hypothesized that the NDI may also play an important role in influencing the efficacy of GIST treatment. However, the effective concentration range of IMA in the Chinese population has not been reported. Recent studies have focused primarily on the IMA, with fewer studies investigating the impact of total IMA + NDI concentrations on GIST patients. Moreover, studies have shown significant individual variability in the pharmacokinetics of IMA, and genetic polymorphisms in metabolic enzymes and transporters involved in drug absorption, distribution, metabolism, and excretion may play crucial roles in these processes. Therefore, the aims of this study were to determine the value of the IMA and total IMA + NDI C_min_ for the prediction of treatment outcomes in advanced GIST patients and to investigate how genetic polymorphisms in metabolic enzymes and transporters affect IMA and NDI concentrations and treatment efficacy, with the goal of providing clinical insights for the use of the IMA in the treatment of advanced GIST.

## 2 Methods

### 2.1 Patients

GIST outpatients who received imatinib mesylate (Gleevec, Novartis, Switzerland) between July 2020 and March 2021 at the First Affiliated Hospital of Soochow University were selected. Upon enrollment, patients were interviewed in person via a self-designed case registration follow-up form to record their name, sex, age, weight, admission diagnosis, IMA dose, total duration of regular medication use up to enrollment, comorbidities, and any concurrent medications. The inclusion criteria were as follows: (1) age ≥18 years; (2) a diagnosis of GIST confirmed by pathological examination (Li et al., 2017); (3) monotherapy with IMA, with normal liver and kidney function before treatment; (4) an IMA treatment duration of ≥28 days, with regular medication use as prescribed, no missed doses in the last 28 days, and no medication taken on the day of follow-up examination; (5) adherence to computed tomography (CT) and other examinations during treatment; and (6) willingness to undergo plasma concentration testing and follow-up 28 days after the test. The exclusion criteria were as follows: (1) used IMA in combination with other antitumor drugs or drugs affecting *CYP3A4* metabolic enzymes; (2) had taken IMA for less than 28 days or had missed doses or interrupted treatment in the last 28 days; (3) were pregnant or lactating; and (4) failed to undergo CT or other required tests during the treatment period. This study was approved by the Ethics Committee of the First Affiliated Hospital of Soochow University. All enrolled patients were informed of the study protocol and provided signed informed consent.

### 2.2 Determination of plasma IMA and NDI concentrations

For each enrolled patient, 4 mL of peripheral venous blood was collected into K_3_-ethylene diamine tetraacetic acid (EDTA) anticoagulant tubes during follow-up. Blood collection was standardized to occur between 22 and 26 h after the last dose of medication. The concentrations of IMA and NDI in the blood samples were then quantified via ultra-performance liquid chromatography–tandem mass spectrometry (UPLC‒MS/MS), as described in our previous publication (Zhang et al., 2022).

### 2.3 DNA extraction and genotyping

For the extraction of genomic DNA from blood samples, the protocol outlined in the instructions provided with the Blood Genomic DNA Rapid Extraction Kit (Sangon, China) was followed. The concentration of the extracted DNA was subsequently determined with a One Drop™ UV Spectrophotometer (Wuyi Technology, China) to evaluate the quality of the obtained genomic DNA. Polymerase chain reaction (PCR) was carried out via T100™ PCR (Bio-Rad, United States). The forward and reverse primer sequences were list in Supplementary Table S1.

### 2.4 Evaluation of treatment effects

According to Chinese consensus guidelines for diagnosis and management of gastrointestinal stromal tumor, contrast-enhanced CT scans should be performed at minimum intervals of 3 months for patients with recurrent/metastatic/unresectable GIST (Li et al., 2017). The effectiveness of IMA treatment was evaluated on the basis of the CT results of GIST patients, and the Choi criteria were used for periodic assessment (Choi, 2008): (1) complete response (CR): all lesions disappeared, and no new tumor lesions appeared; (2) partial response (PR): CT revealed lesion shrinkage of ≥10% or a 15% reduction in the CT value of the tumor, with no new lesions found; (3) stable disease (SD): the maximum diameter of the lesion did not increase enough to meet the criteria for progressive disease (PD), nor did shrinkage meet the criteria for PR; (4) PD: the sum of the largest diameters of the lesions on CT increased by ≥20%, the change in density did not meet the criteria for PR, and new nodules appeared or the volume of existing tumor nodules increased.

### 2.5 Data analysis

Data processing and analysis were conducted via SPSS 26.0 statistical software. Spearman correlation analysis was employed to assess the correlation between different parameters. Comparisons of count data were performed via the *χ* ^*2*^ test. The nonparametric Mann‒Whitney U test was used to compare continuous variables. The predictive and warning values of the IMA and total IMA + NDI trough concentrations for the risk of PD in patients with advanced GIST were analyzed via receiver operating characteristic (ROC) curves. A *P* value of <0.05 was considered to indicate statistical significance.

## 3 Results

### 3.1 Patient characteristics

A total of 21 advanced patients, including 13 (62%) males and 8 (38%) females, with a median age of 60 years (range, 38–76 years), were enrolled, and the IMA and NDI trough concentration tests were completed. The median body mass index (BMI) was 22 kg/m^2^ (range, 20–25 kg/m^2^). The primary GIST site was the intestinal tract in 14 (67%) patients and the stomach in 7 (33%) patients. The daily dose of IMA was 400 mg in 16 (85%) patients and 600 mg in 5 (7%) patients. The mean duration of regular medication was 4.9 years (range, 36 days to 18 years). There were six patients with PD, 14 patients with SD, one patient with a PR, and no patients with a CR during treatment. Advanced GIST patients with PD had significantly lower IMA and IMA + NDI C_min_ values than did those without PD (742 ng/mL vs. 1,684 ng/mL, *P* < 0.001; 936 ng/mL vs. 2050 ng/mL, *P* < 0.001, respectively) (Table 1).

**TABLE 1:** Comparison of clinical characteristics between advanced GIST patients with and without PD (*n* = 21).

Characteristics	With PD (*n* = 6)	Without PD (*n* = 15)	*P*-value
Sex [number (%)]			0.590
Male	4 (67)	9 (60)	
Female	2 (33)	6 (40)	
Age (years)	53 ± 9	62 ± 11	0.063
BMI (kg/m^2^)	21 ± 2	22 ± 3	0.413
Primary tumor site [number (%)]			0.701
Stomach	2 (33)	5 (33)	
Intestinal tract	4 (67)	10 (67)	
Dose [number (%)]			0.550
400 mg/d	5 (83)	11 (73)	
600 mg/d	1 (17)	4 (27)	
C_min_ of IMA (ng/mL)	742 ± 297	1,684 ± 636	<0.001
C_min_ of IMA + NDI (ng/mL)	936 ± 359	2050 ± 655	<0.001
*ABCG2* (rs2231137) [number (%)]			0.007
CC	5 (63)	3 (38)	
CT + TT	1 (8)	12 (92)	
*ABCG2* (rs2725252) [number (%)]			0.038
CC	1 (9)	10 (91)	
CA + AA	5 (50)	5 (50)	
*SLC22A5* (rs2631372) [number (%)]			0.002
GG	5 (71)	2 (29)	
GC + CC	1 (7)	13 (93)	

### 3.2 Effects of genetic polymorphisms on disease status

The genes examined in this study include 18 members of the *CYP450*-metabolizing enzyme family and transporters, in which SNPs have been reported to be potentially relevant in the *in vivo* processing or efficacy of IMA and in the occurrence of adverse effects (Adeagbo et al., 2016; Angelini et al., 2013a; Angelini et al., 2013b; Bouchet et al., 2016; Delord et al., 2013; Di Paolo et al., 2014; Harivenkatesh et al., 2017; Kassogue et al., 2014; Petain et al., 2008; Qiu et al., 2018; Singh et al., 2012; Skoglund et al., 2014; Verboom et al., 2019; Zheng et al., 2015). These genes were as follows: (1) genes encoding *CYP-*metabolizing enzymes, including *CYP1A2* (rs762551), *CYP2B6* (rs3745274), *CYP3A4* (rs2242480), and *CYP3A5* (rs776746); and (2) genes encoding transporters, including *ABCG2* (rs2725252), *ABCG2* (rs2231137), *ABCG3* (rs2231142), *ABCB1* (rs28656907), *ABCB1* (rs1128503), *ABCB1* (rs1045642), *ABCB4* (rs1202283), and *ABCC2* (rs2273697) of the *ABC* family and *SLC22A1* (rs628031), *SLC22A2* (rs683369), *SLC22A5* (rs2631372), *SLC22A5* (rs274558), *SLC19A1* (rs12659), and *SLC19A1* (rs1051266) of the *SLC* family. As shown in Table 1, carriers of the C allele of rs2231137 in *ABCG2*, the A allele of rs2725252 in *ABCG*2, and the G allele of rs2631372 in *SLC22A5* had a greater chance of disease progression (63% vs. 8%, *P* = 0.007; 50% vs. 9%, *P* = 0.038; 71% vs. 7%, *P* = 0.002). For the other 15 selected SNPs, no significant differences were observed between the different disease state groups.

### 3.3 Value of the IMA and IMA + NDI trough concentrations in predicting PD

**FIGURE 1:**
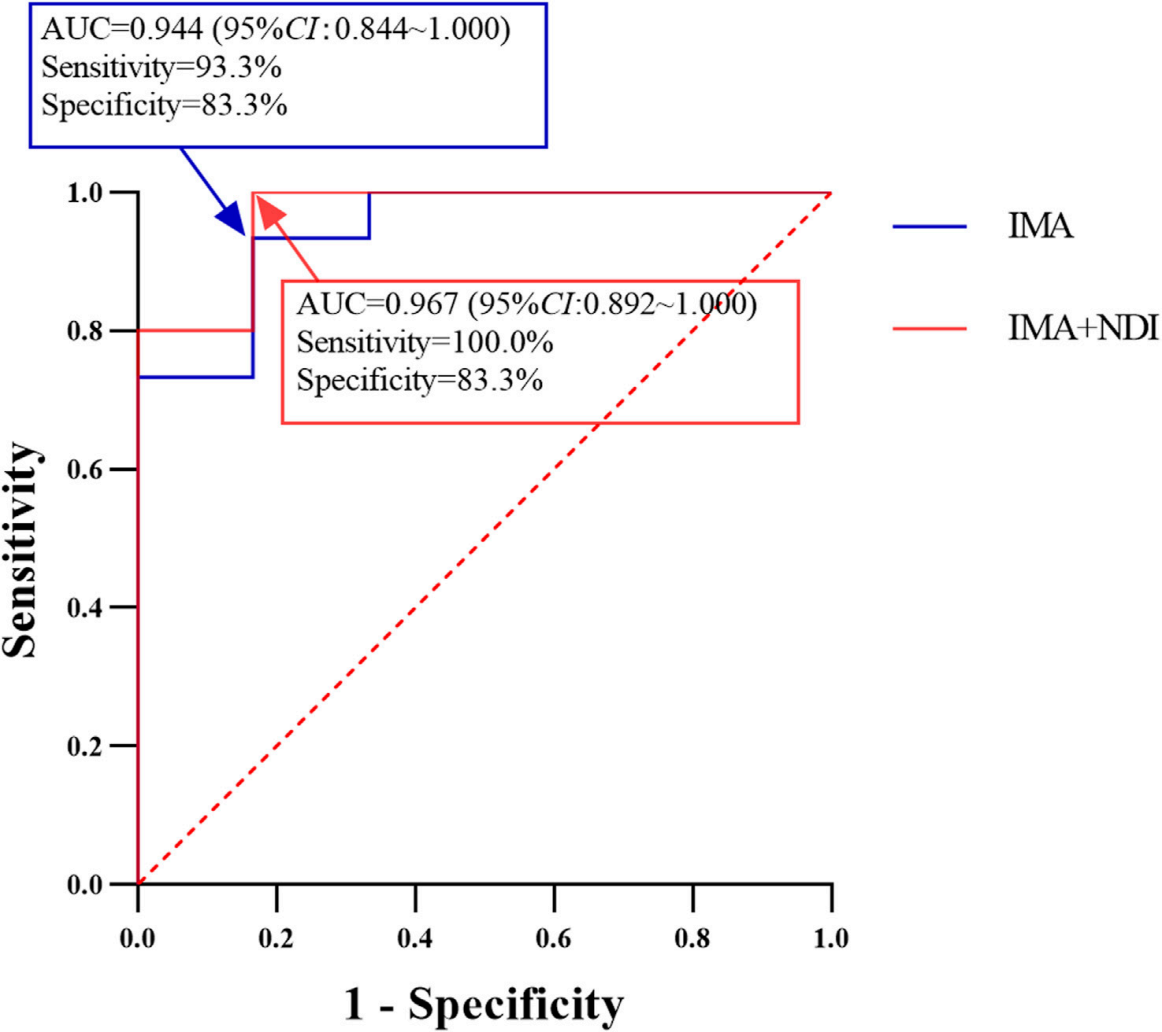
Performance of IMA and IMA + NDI C_min_ cutoff values in predicting the development of PD in patients with advanced GIST (*n* = 21).

ROC curve analysis of the IMA trough concentration revealed an area under the curve of 0.944 (95% confidence interval [CI]: 0.844–1.000) (Figure 1). The cutoff point for predicting the development of PD in advanced GIST patients was 950 ng/mL, with a sensitivity of 93.3% and specificity of 83.3%. Using this cutoff, the 21 GIST patients were divided into two groups: those with IMA trough concentrations ≥950 ng/mL had a PD incidence of 6.7% (1/15), whereas those with IMA trough concentrations <950 ng/mL had a PD incidence of 83.3% (5/6), with a statistically significant difference between the groups (*P* < 0.001).

Similarly, ROC curve analysis of the IMA + NDI trough concentration revealed an area under the curve of 0.967 (95% CI: 0.892–1.000). The cutoff value for predicting the development of PD was 956 ng/mL, with a sensitivity of 100.0% and a specificity of 83.3%. Using this cutoff, the patients were divided into two groups: those with trough concentrations ≥956 ng/mL had a PD incidence of 6.3% (1/16), whereas those with trough concentrations <956 ng/mL had a PD incidence of 100.0% (5/5), with a statistically significant difference between the groups (*P* < 0.001).

### 3.4 Impacts of genetic polymorphisms on IMA and IMA + NDI C_min_

**FIGURE 2:**
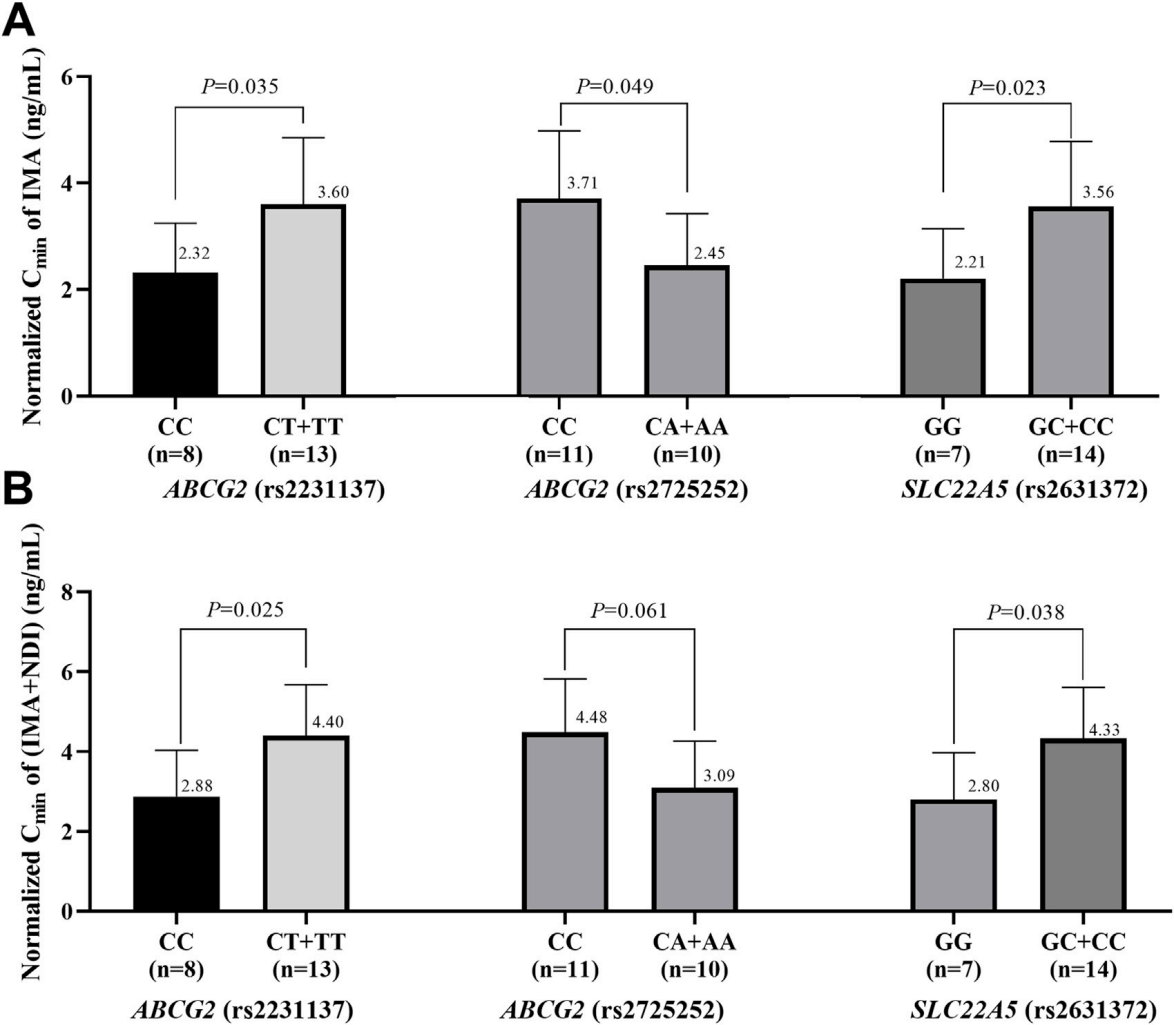
Impacts of gene polymorphisms on the C_min_ of IMA **(A)** and IMA + NDI **(B)**.

The effects of genetic polymorphisms in the three metabolic enzymes and transporters associated with the outcomes of advanced GIST patients on the IMA and total IMA + NDI C_min_ were further analyzed. To account for variations in IMA dosing, the C_min_ values of IMA and NDI were normalized to the trough concentration corresponding to a single milligram of IMA. As shown in Figure 2, IMA and IMA + NDI C_min_ were significantly greater in C allele carriers of rs2231137 in *ABCG2*, A allele carriers of rs2725252 in ABC*G2*, and G allele carriers of rs2631372 in *SLC22A5*. Although no significant difference in IMA + NDI C_min_ was observed for A allele carriers of rs2725252 in *ABCG2*, a trend toward higher concentrations was noted (IMAs: 2.32 ng/mL vs. 3.60 ng/mL, *P* = 0.035; 2.45 ng/mL vs. 3.71 ng/mL, *P* = 0.049; 2.21 ng/mL vs. 3.56 ng/mL, *P* = 0.023; IMA + NDI: 2.88 ng/mL vs. 4.40 ng/mL, *P* = 0.025; 3.09 ng/mL vs. 4.48 ng/mL, *P* = 0.061; 2.80 ng/mL vs. 4.33 ng/mL, *P* = 0.038).

## 4 Discussion

Numerous studies have shown a strong correlation between the C_min_ of the IMA and outcomes in patients with advanced GIST (Bouchet et al., 2016; Demetri et al., 2009; Teranishi et al., 2023). However, no reports have clarified the effective range of the IMA C_min_ in the Chinese population. Moreover, NDI, a metabolite with similar activity to IMA (Al-Hadiya et al., 2014), has rarely been included in safety and efficacy studies. Therefore, investigating the effective concentration ranges of IMA and IMA + NDI in the Chinese population is crucial. Among the 21 advanced GIST patients in this study, the mean IMA and IMA + NDI trough concentrations in the six patients with PD were 742 ± 297 ng/mL and 936 ± 359 ng/mL, respectively, which were significantly lower than those in the non-PD group (1,684 ± 636 ng/mL and 2050 ± 655 ng/mL), indicating the feasibility of using the IMA and total IMA + NDI C_min_ as indicators of efficacy in patients with advanced GIST. The effective ranges of the IMA and total IMA + NDI C_min_ were further analyzed via ROC curves. The results indicated that the risk of PD was significantly reduced in GIST patients with an IMA C_min_ of ≥950 ng/mL or a total IMA + NDI C_min_ of ≥956 ng/mL (*P* < 0.001; *P* < 0.001). These findings suggest that advanced GIST patients derive greater clinical benefits when the C_min_ of the IMA is ≥950 ng/mL or when the C_min_ of the IMA + NDI is ≥956 ng/mL. These thresholds are similar to those reported in a Japanese study, in which maintaining the IMA C_min_ above 917 ng/mL was recommended to increase progression-free survival (PFS) in advanced GIST patients (Teranishi et al., 2023). However, this IMA C_min_ threshold is greater than that reported in a French study, which revealed that an IMA C_min_ greater than 760 ng/mL was associated with prolonged PFS (Bouchet et al., 2016). Furthermore, this IMA C_min_ threshold is lower than the 1,110 ng/mL recommended in a U.S. study (Demetri et al., 2009). These differences suggest that ethnic variation may be an important factor influencing IMA pharmacokinetics.

Substantial intraindividual and interindividual variabilities in IMA blood concentrations have been observed among patients with GIST (Peng et al., 2005). Genes involved in the absorption, distribution, metabolism, and excretion of drugs may significantly influence the pharmacokinetics of IMA. Owing to the presence of polymorphisms in many metabolic enzymes and transporters, we investigated the effects of polymorphisms in 18 IMA-related metabolic enzyme and transporter genes on the efficacy and plasma concentrations of IMA in advanced GIST patients (Adeagbo et al., 2016; Angelini et al., 2013a; Angelini et al., 2013b; Bouchet et al., 2016; Delord et al., 2013; Di Paolo et al., 2014; Harivenkatesh et al., 2017; Kassogue et al., 2014; Petain et al., 2008; Qiu et al., 2018; Singh et al., 2012; Skoglund et al., 2014; Verboom et al., 2019; Zheng et al., 2015). As a result, carriers of the C allele in rs2231137 in *ABCG2*, the A allele in rs2725252 in *ABCG*2, and the G allele in rs2631372 presented a greater risk of disease progression and a lower C_min_ of both IMA and IMA + NDI. These findings suggest that *ABCG2* (rs2231137), *ABCG2* (rs2725252), and *SLC22A5* (rs2631372) polymorphisms may affect treatment outcomes in advanced GIST patients by modulating IMA and NDI concentrations. Therefore, it is recommended to assess genetic polymorphisms in patients prior to treatment. An appropriate IMA dosage can be determined based on the genetic results, and regular monitoring of IMA concentrations is recommended in advanced GIST patients.

A key limitation of this study is the modest sample size (*n* = 21) recruited from a single institution, which may introduce selection bias and constrain the generalizability of the conclusions. Future multi-center studies with larger cohorts are needed to validate these results.

## 5 Conclusion

In summary, the IMA and total IMA + NDI C_min_ can be used as effective indicators for assessing treatment efficacy in Chinese advanced GIST patients. Drug efficacy should be closely monitored in patients with an IMA C_min_ <950 ng/mL or a total IMA + NDI C_min_ <956 ng/mL, and the IMA dosing regimen should be adjusted accordingly to ensure optimal clinical outcomes. Furthermore, genetic polymorphism testing is recommended prior to dosing to appropriately adjust the IMA dose for carriers of the C allele in rs2231137 in *ABCG2*, the A allele in rs2725252 in *ABCG*2, and the G allele in rs2631372. The limitation of this study is the small sample size, which may be statistically biased in the results and will be followed up with further sample size expansion studies.

## Data Availability

The original contributions presented in the study are included in the Supplementary Material, further inquiries can be directed to the corresponding author. The SNP data presented in this study can be found in online repositories: https://www.ncbi.nlm.nih.gov/snp/ rs762551, rs3745274, rs2242480, rs776746, rs2725252, rs2231137, rs2231142, rs28656907, rs1128503, rs1045642, rs1202283, rs2273697, rs628031, rs683369, rs2631372, rs274558, rs12659, and rs1051266.

## References

[B1] Adeagbo B. A. Bolaji O. O. Olugbade T. A. Durosinmi M. A. Bolarinwa R. A. Masimirembwa C. (2016). Influence of CYP3A5*3 and ABCB1 C3435T on clinical outcomes and trough plasma concentrations of imatinib in Nigerians with chronic myeloid leukaemia. J. Clin. Pharm. Ther. 41 (5), 546–551. 10.1111/jcpt.12424 27426203

[B2] Al-Hadiya B. M. Bakheit A. H. Abd-Elgalil A. A. (2014). Imatinib mesylate. Profiles Drug Subst. Excip. Relat. Methodol. 39, 265–297. 10.1016/B978-0-12-800173-8.00006-4 24794909

[B3] Angelini S. Pantaleo M. A. Ravegnini G. Zenesini C. Cavrini G. Nannini M. (2013a). Polymorphisms in OCTN1 and OCTN2 transporters genes are associated with prolonged time to progression in unresectable gastrointestinal stromal tumours treated with imatinib therapy. Pharmacol. Res. 68 (1), 1–6. 10.1016/j.phrs.2012.10.015 23127916

[B4] Angelini S. Soverini S. Ravegnini G. Barnett M. Turrini E. Thornquist M. (2013b). Association between imatinib transporters and metabolizing enzymes genotype and response in newly diagnosed chronic myeloid leukemia patients receiving imatinib therapy. Haematologica 98 (2), 193–200. 10.3324/haematol.2012.066480 22875622 PMC3561425

[B5] Bouchet S. Poulette S. Titier K. Moore N. Lassalle R. Abouelfath A. (2016). Relationship between imatinib trough concentration and outcomes in the treatment of advanced gastrointestinal stromal tumours in a real-life setting. Eur. J. Cancer 57, 31–38. 10.1016/j.ejca.2015.12.029 26851399

[B6] Cavnar M. J. Seier K. Curtin C. Balachandran V. P. Coit D. G. Yoon S. S. (2021). Outcome of 1000 patients with gastrointestinal stromal tumor (GIST) treated by surgery in the pre- and post-imatinib eras. Ann. Surg. 273 (1), 128–138. 10.1097/SLA.0000000000003277 30946076 PMC6774913

[B7] Choi H. (2008). Response evaluation of gastrointestinal stromal tumors. Oncologist 13 (Suppl. 2), 4–7. 10.1634/theoncologist.13-S2-4 18434631

[B8] Delbaldo C. Chatelut E. Re M. Deroussent A. Seronie-Vivien S. Jambu A. (2006). Pharmacokinetic-pharmacodynamic relationships of imatinib and its main metabolite in patients with advanced gastrointestinal stromal tumors. Clin. Cancer Res. 12 (20 Pt 1), 6073–6078. 10.1158/1078-0432.CCR-05-2596 17062683

[B9] Delord M. Rousselot P. Cayuela J. M. Sigaux F. Guilhot J. Preudhomme C. (2013). High imatinib dose overcomes insufficient response associated with ABCG2 haplotype in chronic myelogenous leukemia patients. Oncotarget 4 (10), 1582–1591. 10.18632/oncotarget.1050 24123600 PMC3858547

[B10] Demetri G. D. Wang Y. Wehrle E. Racine A. Nikolova Z. Blanke C. D. (2009). Imatinib plasma levels are correlated with clinical benefit in patients with unresectable/metastatic gastrointestinal stromal tumors. J. Clin. Oncol. 27 (19), 3141–3147. 10.1200/JCO.2008.20.4818 19451435

[B11] Di Paolo A. Polillo M. Capecchi M. Cervetti G. Barate C. Angelini S. (2014). The c.480C>G polymorphism of hOCT1 influences imatinib clearance in patients affected by chronic myeloid leukemia. Pharmacogenomics J. 14 (4), 328–335. 10.1038/tpj.2014.7 24589908

[B12] Harivenkatesh N. Kumar L. Bakhshi S. Sharma A. Kabra M. Velpandian T. (2017). Do polymorphisms in MDR1 and CYP3A5 genes influence the risk of cytogenetic relapse in patients with chronic myeloid leukemia on imatinib therapy? Leuk. Lymphoma 58 (9), 1–9. 10.1080/10428194.2017.1287359 28367681

[B13] Heinrich M. C. Rankin C. Blanke C. D. Demetri G. D. Borden E. C. Ryan C. W. (2017). Correlation of long-term results of imatinib in advanced gastrointestinal stromal tumors with next-generation sequencing results: analysis of phase 3 SWOG intergroup trial S0033. JAMA Oncol. 3 (7), 944–952. 10.1001/jamaoncol.2016.6728 28196207 PMC5727908

[B14] Kassogue Y. Quachouh M. Dehbi H. Quessar A. Benchekroun S. Nadifi S. (2014). Functional polymorphism of CYP2B6 G15631T is associated with hematologic and cytogenetic response in chronic myeloid leukemia patients treated with imatinib. Med. Oncol. 31 (1), 782. 10.1007/s12032-013-0782-6 24293093

[B15] Li J. Ye Y. Wang J. Zhang B. Qin S. Shi Y. (2017). Chinese consensus guidelines for diagnosis and management of gastrointestinal stromal tumor. Chin. J. Cancer Res. 29 (4), 281–293. 10.21147/j.issn.1000-9604.2017.04.01 28947860 PMC5592117

[B16] Peng B. Lloyd P. Schran H. (2005). Clinical pharmacokinetics of imatinib. Clin. Pharmacokinet. 44 (9), 879–894. 10.2165/00003088-200544090-00001 16122278

[B17] Petain A. Kattygnarath D. Azard J. Chatelut E. Delbaldo C. Geoerger B. (2008). Population pharmacokinetics and pharmacogenetics of imatinib in children and adults. Clin. Cancer Res. 14 (21), 7102–7109. 10.1158/1078-0432.CCR-08-0950 18981009

[B18] Poveda A. Garcia Del Muro X. Lopez-Guerrero J. A. Cubedo R. Martinez V. Romero I. (2017). GEIS guidelines for gastrointestinal sarcomas (GIST). Cancer Treat. Rev. 55, 107–119. 10.1016/j.ctrv.2016.11.011 28351781

[B19] Qiu H. B. Zhuang W. Wu T. Xin S. Lin C. Z. Ruan H. L. (2018). Imatinib-induced ophthalmological side-effects in GIST patients are associated with the variations of EGFR, SLC22A1, SLC22A5 and ABCB1. Pharmacogenomics J. 18 (3), 460–466. 10.1038/tpj.2017.40 28762371

[B20] Raut C. P. Espat N. J. Maki R. G. Araujo D. M. Trent J. Williams T. F. (2018). Efficacy and tolerability of 5-year adjuvant imatinib treatment for patients with resected intermediate- or high-risk primary gastrointestinal stromal tumor: the PERSIST-5 clinical trial. JAMA Oncol. 4 (12), e184060. 10.1001/jamaoncol.2018.4060 30383140 PMC6440723

[B21] Reichardt P. (2018). The story of imatinib in GIST - a journey through the development of a targeted therapy. Oncol. Res. Treat. 41 (7-8), 472–477. 10.1159/000487511 29895025

[B22] Singh O. Chan J. Y. Lin K. Heng C. C. Chowbay B. (2012). SLC22A1-ABCB1 haplotype profiles predict imatinib pharmacokinetics in Asian patients with chronic myeloid leukemia. PLoS One 7 (12), e51771. 10.1371/journal.pone.0051771 23272163 PMC3525665

[B23] Skoglund K. Boiso Moreno S. Jonsson J. I. Vikingsson S. Carlsson B. Green H. (2014). Single-nucleotide polymorphisms of ABCG2 increase the efficacy of tyrosine kinase inhibitors in the K562 chronic myeloid leukemia cell line. Pharmacogenet Genomics. 24 (1), 52–61. 10.1097/FPC.0000000000000022 24322003

[B24] Teranishi R. Takahashi T. Nishida T. Kurokawa Y. Nakajima K. Koh M. (2023). Plasma trough concentration of imatinib and its effect on therapeutic efficacy and adverse events in Japanese patients with GIST. Int. J. Clin. Oncol. 28 (5), 680–687. 10.1007/s10147-023-02325-x 36971916

[B25] Verboom M. C. Kloth J. S. L. Swen J. J. Sleijfer S. Reyners A. K. L. Steeghs N. (2019). Genetic polymorphisms in ABCG2 and CYP1A2 are associated with imatinib dose reduction in patients treated for gastrointestinal stromal tumors. Pharmacogenomics J. 19 (5), 473–479. 10.1038/s41397-019-0079-z 30713339

[B26] Zhang M. Liu X. Chen Z. Jiang S. Wang L. Tao M. (2022). Method development and validation for simultaneous determination of six tyrosine kinase inhibitors and two active metabolites in human plasma/serum using UPLC-MS/MS for therapeutic drug monitoring. J. Pharm. Biomed. Anal. 211, 114562. 10.1016/j.jpba.2021.114562 35124453

[B27] Zheng Q. Wu H. Yu Q. Kim D. H. Lipton J. H. Angelini S. (2015). ABCB1 polymorphisms predict imatinib response in chronic myeloid leukemia patients: a systematic review and meta-analysis. Pharmacogenomics J. 15 (2), 127–134. 10.1038/tpj.2014.54 25245580

