# Supplementary material for: Study of genetic polymorphisms and steady-state trough concentrations of imatinib and its active metabolite in predicting efficacy in gastrointestinal stromal tumors
Source: Front Pharmacol. 2025 May 19;16:1604619. doi: 10.3389/fphar.2025.1604619 (PMC12127160; doi:10.3389/fphar.2025.1604619)
Supplement: Supplementary file 1 [file Table1.docx]

Supplementary Table S1: The forward and reverse primer sequences of 18 members of the *CYP450*-metabolizing enzyme family and transporters.

Table S1. Sequence of PCR primers

| Name | Forward primer (5'-3') | Reverse primer (5'-3') |
| --- | --- | --- |
| [*CYP1A2*](https://www.pharmgkb.org/gene/PA27093) (rs762551) | TCCCAACTCTCTCCATAGGAAGAAG | TGGAGCACATCCTTCCATTGTAATC |
| *CYP2B6* (rs3745274) | TTTGCAGAGATAAACCAATAAATCC | GACTATTATGCCAAAACTGTTCACC |
| [*CYP3A4*](https://www.pharmgkb.org/gene/PA130) (rs2242480) | TACCTGCCTTCAATTTTTCACTGAC | CTAGTTCATTAGGGTGTGACACACA |
| [*CYP3A5*](https://www.pharmgkb.org/gene/PA131) (rs776746) | AGAGAGCCTATGAATGGACGTGCGG | GGAAATAGGACGTGAAGATAGACAA |
| *ABCG2* (rs2725252) | GGGAGGCCTGCAGACCATCTTCCAA | GTGATGAAGCTCTCCCCTGGCCGTA |
| *ABCG2* (rs2231137) | GTATTGTCACCTAGTGTTTGCAATC | TTCTCAACTGGTTTTCGACAAGGTA |
| *ABCG3* (rs2231142) | CAGTCATGGTCTTAGAAAAGACTCA | ACACAGGGAAAGTCCTACTTATGCT |
| *ABCB1* (rs28656907) | GAACAGTCAGTTCCTATATCCTGTG | TTGAAAGGGCAACATCAGAAAGATG |
| *ABCB1* (rs1128503) | CTGGTCCTGAAGTTGATCTGTGAAC | TCCCAGGCTGTTTATTTGAAGAGAG |
| *ABCB1* (rs1045642) | CTGGTCGACAGATCAGGAAAATTAG | TAGGCTATAGATGCTGCTAGACATG |
| [*ABCB4*](https://www.pharmgkb.org/gene/PA267) (rs1202283) | TAGACAAAGGTAGCACCTCATCTTT | ACCTCTACCTGGATTTCCTTTACTC |
| *ABCC2* (rs2273697) | AGAAACTGCAGGAGGAAATTGATGC | TAATAGAAAGCAGATGAACCAGAGC |
| *SLC22A1* (rs628031) | CCTTTACATACCTGTCTGCAAAGTA | TAGTAGGAGAAAACTCTGTGAAACA |
| *SLC22A2* (rs683369) | TTTTGAGTGTGGAGAAAACATTCTG | TTTATGCATTTGGCTACATGGTGCT |
| *SLC22A5* (rs2631372) | GTACCTAACTTTAAATCTCCCTAGA | AGGAACAGGAGACATTTCTCTGAAT |
| *SLC22A5* (rs274558) | AAAATAAAGCTACAGAAAGGGAACC | AGCTTTGCTTTCTTTTTCAGTTAGT |
| *SLC19A1* (rs12659) | AGTCTGGTAGAACAAGTTCAGCATC | GTGCTGGTACATAATTAGCTGTTAC |
| *SLC19A1* (rs1051266) | GCAGCCTCTTCTTCAACCGCGACGA | GTTCCACAGGATGTGCACGTAGTAG |
